# Assessing differentiation in cutaneous squamous cell carcinoma: A machine learning approach

**DOI:** 10.1016/j.jdin.2025.07.004

**Published:** 2025-08-05

**Authors:** Victor Liang, Elias Engström, Martin Gillstedt, John Paoli, Magdalena Claeson, Isabelle Krakowski, Kari Nielsen, Jenna Pakka, Niki Radros, Mari Salmivuori, Sam Polesie

**Affiliations:** a Department of Dermatology and Venereology, Institute of Clinical Sciences, Sahlgrenska Academy, University of Gothenburg, Gothenburg, Sweden; b Region Västra Götaland, Sahlgrenska University Hospital, Department of Dermatology and Venereology, Gothenburg, Sweden; c Department of Oncology and Pathology, Karolinska Institute, Stockholm, Sweden; d Department of Dermatology/Theme Inflammation, Karolinska University Hospital, Stockholm, Sweden; e Lund University Cancer Center, LUCC and Lund University Skin Cancer Research Group, LUSCaR Lund, Sweden; f Division of Dermatology, Skåne University Hospital and Department of Clinical Sciences, Faculty of Medicine, Lund University, 22185, Lund, Sweden; g Theme Cancer, Karolinska University Hospital, Stockholm, Sweden; h Department of Dermatology and Allergology, University of Helsinki and Helsinki University Hospital, Helsinki, Finland

**Keywords:** artificial intelligence, convolutional neural network, machine learning, squamous cell carcinoma

*To the Editor*: Cutaneous squamous cell carcinomas (cSCC) comprise a significant proportion of all cancers managed by dermatologists.^1^ Although a clinical diagnosis of cSCC in most cases is uncomplicated, preoperative determination of the degree of differentiation is often more challenging. Nevertheless, an accurate estimation of tumor differentiation is important for surgical planning and margin determination.^2^ Moreover, well-differentiated cSCC, may be suitable for nonsurgical treatments including curettage, electrodessication, or shave removal in selected patient populations.^3^ The aim of this study was to examine how a convolutional neural network (CNN) performed in discriminating between well vs moderately and poorly differentiated cSCCs.

In this retrospective investigation, we trained a de novo CNN on 1,829 clinical close-up images of cSCCs (Supplemental Material, available via Mendeley at https://doi.org/10.17632/p39x5jzjf3.1). Among these 1254 (68.6%) were well-differentiated, and 575 (31.4%) were moderately or poorly differentiated.

All images were obtained from the Department of Dermatology, in Gothenburg, Sweden over a nine-year period (2015 to 2023) and were randomized into a training (*n =* 1,329), validation (*n =* 200), and test set (*n =* 300, Supplemental Material). The CNN’s performance was compared to the combined assessment of seven independent readers, one senior resident and six board-certified dermatologists with experience ranging from 4 to 25 years (median 10 years). Readers were instructed to provide their evaluations, specify their certainty level with respect to the level of differentiation, and evaluate the presence of predefined clinical features for each case (Supplemental Material). Interobserver agreement was assessed using kappa (κ) statistics.

The CNN model yielded a receiver operating characteristic curve with an area under the curve of 0.69 (95% CI, 0.63-0.76). The corresponding area under the curve for the combined dermatologists’ assessment was 0.70 (95% CI; 0.64-0.76, *P* = .79) (Fig 1).

**Fig 1:**
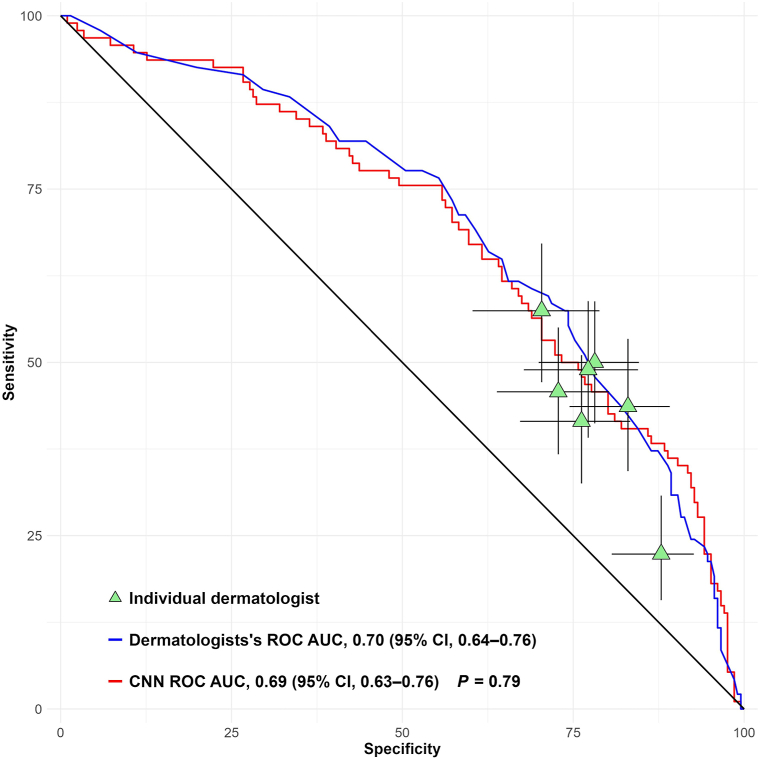
ROC curves and AUC comparison of CNN model vs dermatologists for assessment of cSCC differentiation. *P* value calculated with DeLong’s test comparing AUCs. *AUC*, Area under the curve; *CNN*, convolutional neural network; *cSCC*, cutaneous squamous cell carcinoma; *ROC*, receiver operating characteristic.

The interobserver agreement in the assessment of cSCC differentiation between the readers was moderate (κ = 0.44, 95% CI, 0.39-0.49). Overall, interobserver agreement for clinical features ranged from fair to substantial (Supplemental Material).

Ulceration and flat surface topography were more common in moderately or poorly differentiated tumors, with odds ratios of 2.34 (95% CI, 1.16-4.72) and 2.94 (95% CI, 1.23-7.01), respectively (Fig 2).

**Fig 2:**
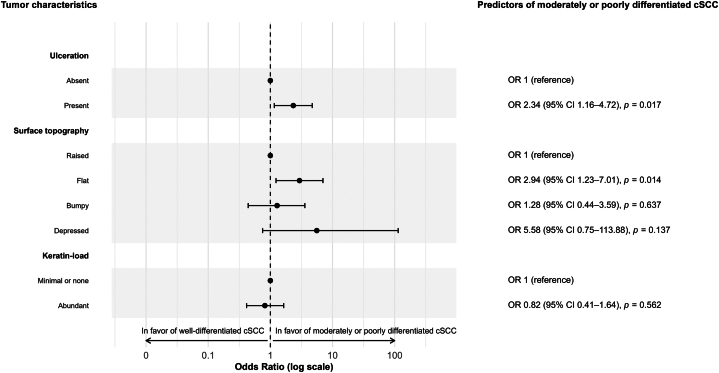
Odds ratio for tumor characteristics. Majority vote of ≥4 dermatologists in agreement of each tumor characteristics to be valid. Higher odds ratios indicate a stronger association with moderately or poorly differentiated cSCC. *OR*, Odds ratio; *cSCC*, cutaneous squamous cell carcinoma.

This study relied on the evaluation of clinical close-up images and it is likely that integration of dermatoscopy images could have produced different results. In real-world practice, physicians integrate additional metadata, such as patient history and dermatoscopic findings, which enhance decision-making compared to assessments made in an artificial setting.^4^^,^^5^ Moreover, the skin tumors analyzed in our study were primarily derived from patients with Fitzpatrick skin types I to III. This investigation, aimed at identifying a novel targeted application for machine learning, demonstrates how a simple artificial-intelligence model performs on par with dermatologists.

Before a CNN model can effectively assist dermatologists in the classification problem of cSCC differentiation in clinical practice, it requires further refinement and prospective evaluation. We believe the use-case presented here is a promising machine learning approach worth pursuing, as it has the potential to assist and augment dermatologists in preoperative decision-making—helping to determine appropriate surgical margins or assess whether alternative, less invasive treatments might be suitable.

## Conflicts of interest

None disclosed.
